# An ethnobotanical survey of medicinal plants used by traditional healers for the treatment of cancer in Hammanskraal and Winterveld, Tshwane Metropolitan Municipality, South Africa

**DOI:** 10.4314/ahs.v21i4.31

**Published:** 2021-12

**Authors:** Idris O Raimi, Boikanyo G Kopaopa, Liziwe L Mugivhisa, Francis B Lewu, Stephen O Amoo, Joshua O Olowoyo

**Affiliations:** 1 Department of Biology, Sefako Makgatho Health Sciences University, P.O. Box 139, Medunsa 0204, South Africa; 2 Department of Agriculture, Cape Peninsula University of Technology, Wellington Campus, Wellington 7655, Western Cape, South Africa; 3 Agricultural Research Council, ARC-Roodeplaat Vegetable and Ornamental Plants, Private Bag X293, Pretoria, 001, South Africa

**Keywords:** Cancer, Indigenous plants, Medicinal plants, South Africa, Traditional practitioners

## Abstract

**Background:**

Knowledge of medicinal plants used by the traditional healers are mostly confined among the locals and the adherents, hence, proper enquiry and documentation can help the ever dynamic scientific world to find permanent cure to the menace of such deadly diseases such as cancer. This study aimed at (1) specifically recording medicinal plants traditionally used for the treatment of cancer in Hammanskraal and Winterveld, South Africa, and (2) documenting the different methods of preparation and administration of those medicinal plants as recorded during the study.

**Method:**

An open-ended semi-structured questionnaire was administered to 90 willing traditional healers in Hammanskraal and Winterveld area, Tshwane Municipality, South Africa to document plants used for cancer treatments. The study was conducted over a period of six months (July – December, 2018). Descriptive statistics was used to present the obtained data.

**Results:**

The study recorded twenty-eight plant species belonging to 18 families for the treatment of different types of cancer. Plant species in the Fabaceae family particularly *Lessertia frutescens* (L.) Goldblatt and J.C. Manning, *Senna italica* Mill and *Trifolium pratense* L. were the most prominently mentioned (highest citation frequency) by the traditional healers for lung, and skin cancer treatment. Based on the citation frequency, the most treated cancer by the traditional healers is in the order: skin cancer > lung cancer > breast cancer > prostate cancer > cervical cancer. The method of preparation included decoction (32.3%), infusion (29%), paste (16.1%) and maceration (22.6%).

**Conclusion:**

In addition to the documentation of indigenous knowledge related to the use of medicinal plants in the traditional management of cancer in South Africa, this study opens a vista for investigations into the phytochemical and pharmacological properties of the documented plants.

## Introduction

Cancer is a serious health challenge rated as the second leading cause of death worldwide next to cardiovascular diseases^1^. The International Agency for Research on Cancer indicated that there were 18.1 million new cases and 9.6 million deaths in 2018, up from the estimated 14.1 million new cancer cases and 8.2 million cancer-related deaths in 2012^2^. Projections based on the Global Burden of Cancer study indicate a substantive increase to over 29 million new cancer cases by year 2040, due to population growth and other social and economic factors^3^. Torre et al.^4^ stated that out of all the new cancer cases globally, over 55% were from developing countries. This scenario plays out because of the low quality of healthcare management system and lack of resources to combat the incidences^5^.

Herbal medicines are prepared using plant parts such as the root, stems, barks, leaves, flowers, fruits or the seeds, harvested from the wild or conservation parks. Africa's richness in cultural and floral diversity provides the necessary recipe for development of new drugs either traditionally or by modern medicine^6^. Conservatively, about 90% of the African population particularly the rural dwellers are still dependent on traditional medicine^7^ and Southern Africa is known to have a large number of indigenous and useful medicinal plants, which have ben used as alternative medicine for many decades^8^. World Health Organization (WHO) shows a livid desire and encourages the documentation of medicinal plants used in treating diseases by natives from different parts of the world^9^. Despite the long history of cancer treatment using herbal remedies in South Africa, there is scarcity of information recording these medicinal plants in Hammanskraal and Winterveld area of Tshwane Metropolitan district. Therefore, documenting these plants becomes imperative so as not to lose vital information resulting from the demise of the traditional healers and also to ignite further scientific exploration.

In this study, we documented the plant species (indigenous and alien) used in the treatment of cancer by traditional healers specifically, Tswana speaking population that resides in Hammanskraal and Winterveld, Tshwane Metropolitan Municipality, South Africa with a view to providing baseline data for future studies into the phytochemical and pharmacological potential of these plants in relation to the management of cancer. The main objectives were to (1) specifically investigate and record medicinal plants used for the treatment of cancer in Hammanskraal and Winterveld, and (2) document the methods of preparation and administration of these medicinal plants during the treatment of different types of cancer.

## Methodology

### Study Area

This study was done in Hammanskraal and Winterveld, Tshwane Metropolitan Municipality, South Africa. Hammanskraal is situated in the northern part of the Gauteng Province, which lies in the latitude 25° 41″ S and longitude 28° 26″ E. Hammanskraal is a large rural area dominated by farms and small holdings with settlers from different Provinces but largely Tswana speaking^10^. On the other hand, Winterveld is located in northwest part of Pretoria and forms part of the Tshwane Metropolitan Municipality. Winterveld lies in the latitude 25° 42″ S and longitude 27° 95″ E10. Winterveld was designated as an apartheid relocation settlement for Bophuthatswana population of South Africa^10^. The study was conducted over a period of six months (July – December, 2018).

### Ethnobotanical and Identification Methods

Information was sourced from the traditional healers through oral interview with some structured questions administered as questionnaires after several reconnaissance visits. The total number of traditional healers per study site was 45. The questions focused on medicinal plants used by the local people from the study sites for the treatment of various cancer types and number of years of traditional healing practice by each practitioner. Furthermore, information about the plant parts used, recipe preparation and methods of recipe administration were documented during the study. Informed consent of the traditional healers who participated in the interview was obtained, including the usage of information given with respect to this study. Plants were collected for voucher number assignment after obtaining collecting permit. Plants collection trip involved the research team, traditional healers and Mr Petros (a Botanist) from the University of Pretoria. Voucher number assignment was done by Magda Nel and the voucher specimens deposited at the H.G.W.J. Schweickerdt Herbarium, University of Pretoria.

### Statistical analysis

Data were presented using descriptive statistics such as percentages and frequencies. Frequency index of each plant species was calculated using the formula:

FI = FC/N × 100

Where FI is % of frequency of citation for one plant species by respondents (traditional practitioners), FC is the number of traditional practitioners who cited the use of a particular plant species, and N is the number of traditional practitioners^11^.

## Results and Discussion

### Interviews with Traditional Practitioners

A total number of ninety traditional healers (45 per township) who were willing to provide information on their scope of work and ready to share their operational methods responded to the questionnaires. Seventy-five percent (75%) of the traditional healers in both locations were females and 25% were males. This is in consonance to the reports of Benarba et al.^12^ and Tchicaillat-Landou et al.^13^ in their ethnobotanical study on medicinal plants used by traditional healers for the treatment of oxidative stress-related diseases in Congo Basin and Algeria where females dominated the gender of the traditional healers. Sixty-three percent (63%) of the traditional healers in our study were between the ages 40 years and above with over fifteen years' practice experience. Tchicaillat-Landou et al.^13^ also reported the ages of the traditional healers interviewed in their ethnobotanical study to be between 40 and 70 years. The ages of the remaining 37% traditional healers were below 40 years with less than 10 years' experience suggesting less interest in traditional practice by the younger generation.

### Plant Frequency index, Habits and Parts Mostly Used for Cancer Treatment

**Figure 1:**
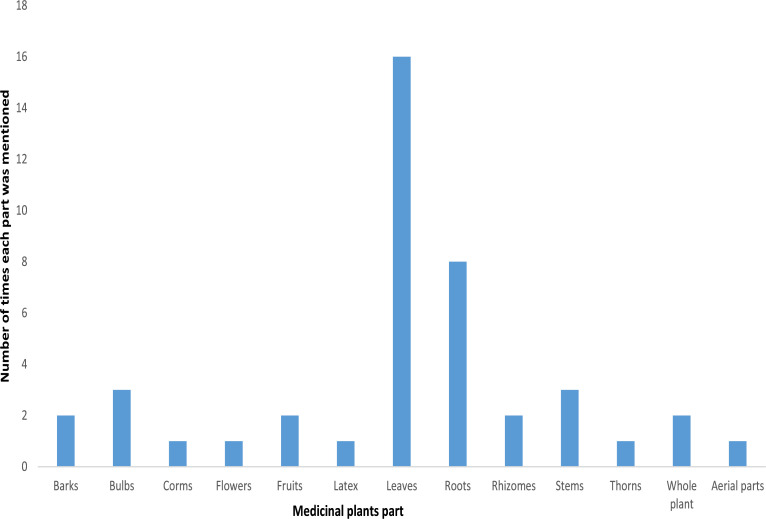
Plant parts used in the herbal preparation

*Lessertia frutescens* (Fabaceae), recorded the highest frequency index i.e. it is the mostly used plant species by the traditional healers in the study locations for skin and lung cancer treatment. It has a long history of usage in the treatment of various diseases according to the folklore medicine^14^. The plants least mentioned were *Euphorbia ingens* E. Mey. ex Boiss., *Justicia* sp var. *capensis* Thunb., and *Asparagus laricinus* Burch (Table 1). Based on the documented plants and the citation frequency during the study, plants habit was in the order: climber < trees < bulbs < herbs = shrubs. Shrubs and herbs were more favored by the traditional healers over other plant habits, which may be linked to the ever present status of shrubs and herbs throughout the season of the year. In agreement to the above assertion, factors such as availability throughout the season of the year, shape, smell, color and size of plants (shrubs and herbs) were attributed to the choice of plants used for treatment in the study of Maema et al.^15^. The plant parts mostly used in the therapeutic preparations by the traditional practitioners were leaves > roots > bulbs = stems > barks > fruits (Table 1; Figure 1). Several ethnobotanical surveys have reported leaves as the mostly used plant parts for treatment of diseases by locals^13^,^16^. For example, Tchicaillat-Landou et al.^13^ in their survey on treatment of oxidative stress by traditional practitioners in Congo noted the leaves as the mostly used plant part. Similarly, Savikin et al.^16^ reported that leaves were the mostly used plant part in their ethnobotanical study in South-western Serbia. Thorns, latex, flowers, and corms were rarely used for recipe during cancer treatment (Figure 1).

**Table 1:** Local/scientific names, types and preparation of plants used for the treatment of cancer in Hammanskraal and Winterveld, Tshwane Municipality, South Africa

Scientific name(s)	Local name(s)/Common name(s)	Voucher Number (PRU)	Family name(s)	Plant Frequency Citation (FC)	Frequency index (FI)	Parts used	Preparation and usage	Type(s) of cancer
***Antizoma*** ***augustifolia* Burch.**		PRU124551	Menispermaceae	3	2.5%	Leaves, Stem	boiled for drinking	Lung cancer, Skin cancer
***Vachellia karroo* (Hayne)** **Banfi & Galasso**	Morumosetlha	PRU0124971	Fabaceae	8	6.8%	Thorn	Dry and use it to mark around the cancer cells	Skin and breast cancer
***Laggera decurrens* (Vahl)** **Hepper & J.R.I Wood**	Wolbos, Pietbos, Bitterbos	PRU 124558	Asteraceae	4	3.4%	Leaves	Leaves are boiled for drinking	Lung cancer, stomach cancer and Skin cancer
***Agapanthus*** ***praecox*** Willd.	Segwana, Kleinblo ulelei (African lily)	PRU0124973	Agapanthaceae	4	3.4%	Bark, roots	Dried and pulverized into powder and taken orally with warm water	Internal cancer
***Asparagus*** ***suaveolens*** Burch	Lesitwane, Makan angwane	PRU124549	Asparagaceae	4	3.4%	Whole plant	Dried bark and roots are pulverized into powder and taken orally with warm water	All cancer types
***Bulbine*** ***narcissifolia* Salm-Dyck**	Monato, Wildesering	PRU124560	Asphodelaceae	4	3.4%	Leaf, roots	Decoction of root. Cold infusion of leaf	Internal cancer and other infections
***Carissa bispinosa* (L.)** **Desf. ex Brenan**	Simboka	PRU124547	Apocynaceae	5	4.2%	Roots, leaves, fruits	Dried, crushed into a powder mixture. The mixture is used to make paste.	Breast cancer, Prostate cancer, cervical cancer
***Centella asiatica* (L.) Urb.**	Mokgwapha/Sekg ophana Kola	PRU0124965	Apiaceae	3	2.5%	Whole plant	Boiled fresh roots for drinking. Stems and dry leaves are crushed and apply on the skin	Skin cancer
***Combretum*** ***mkuzense* J.D.Carr &** **Retief**	Motshwere, Mutswiri, Moduba	PRU0124959	Combretaceae	3	2.5%	Roots	Grinded into a paste and used as lotion.	Skin, breast
***Convolvulus*** ***saggitatus* Thunb.**	Bindweed	PRU124546	Convolvulaceae	2	1.7%	Leaves, Aerial part	Alcohol extracts are taken orally	Lung cancer, Skin cancer, Prostate cancer
***Conyza podocephala* DC.**		PRU0124962	Asteraceae	2	1.7%	Roots	Fresh roots are boiled for drinking	Lung cancer, Stomach cancer
***Asparagus*** ***laricinus* Burch.**		PRU0124969	Asparagaceae	1	0.8%	Bulb	Boiled and used for blood cleansing to flush out the pathogens	Skin cancer, Cervical cancer and Prostate cancer
***Eucomis*** ***autumnalis* (Mill.) Chit.**	Pineapple lily, Umathunga	PRU0124952	Asparagaceae	4	3.4%	Leaves, Roots	Dried roots are crushed into powder and infused into water for drinking	Oesophageal, lung cancer
***Euphorbia ingens* E. Mey.** **ex Boiss**	Nkonde, Motlalamaswi	PRU0124951	Euphorbiaceae	1	0.8%	Latex	Apply latex on the affected skin	Skin, breast cancer
***Felicia muricata* Thunb.**	Kapa Bokone, Mooka	PRU124556	Asteraceae	5	4.2%	Leaves	Leaves are boiled and administered orally	Internal cancer, Inflammation
***Hypoxis*** ***hemerocallidea* (Fisch. &** **C.A. Mey.)**	Lotsane, sterblom gifbol	PRU0124953	Hypoxidaceae	4	3.4%	Corms	Dried and boiled for drinking	Skin cancer, Lung cancer, Cervical, Prostate cancer
***Hypoxis iridifolia* Baker**		PRU124554	Hypoxidaceae	3	2.5%	Rhizomes	Plant decoctions and administered orally	Lung cancer, Internal cancer
***Justicia sp.* var.** ***capensis* (Thunb.)**	Khokhela, Mooka, Rooiwortelboom, Soetdoring, Mooka	PRU124559	Acanthaceae	1	0.8%	Leaves, Roots	Leaf extracts and taken orally, dry the roots and crush into powder and thereafter pour into hot water	Lung cancer, Breast cancer
***Lessertia frutescens* (L.)**	Musapelo, phetola	PRU0124957	Fabaceae	13	11.0%	Leaves	Leaves are boiled and administered orally	Skin cancer, Lung cancer
***Merwilla*** ***plumbea* (Lindl.) Speta**	Morutlhare, Pitsae- kgolo	PRU0124954	Hyacinthaceae	3	2.5%	Bulbs	Leaves macerated and applied on the affected skin parts	Skin cancer
***Pittosporum*** ***viridiflorum* (Sims.)**	Nkasur, Umkhwenke	PRU0124958	Pittosporaceae	6	5.1%	Bark, roots	Bark and roots boiled and used for steam bathing	Breast, skin cancer
***Sansevieria*** ***aethiopica* Thunb.**	Mogodiri, Maroon honey flower	PRU124552	Asparagaceae	4	3.4%	Leaves	Alcohol leaves extraction and administered orally	Internal cancer
***Dietes grandiflora* N.E.Br.**		PRU0124968	Iridaceae	3	2.5	Leaves, flowers,rhizome	Leaves, flowers and rhizomes crushed, thereafter boiled and administered orally	Lung cancer, Cervical cancer, Skin cancer
***Senna italica* Mill.**	Black Storm, Rumo lamadi	PRU124557	Fabaceae	8	6.8%	Leaves	leaves shade dried and pulverized to powder in a mechanical grinder and administered orally	Internal cancer
***Trifolium pratense* (L.)**	Rooiklawer, swartklawer	PRU0124960	Fabaceae	7	5.9%	Leaves	Leaves boiled and administered orally	All cancer types
***Tulbaghia*** ***violacea* (Harv.)**	Wild garlic	PRU0124956	Alliaceae	4	3.4%	Bulbs, Leaves	Bulbs and leaves eaten fresh. Leaves crushed and boiled for oral administration	Oesophageal cancer
***Solanum aculeastrum* (L.)**	Mokgopa	PRU124555	Solanaceae	5	4.2%	Leaves, stem	Leaves and stems boiled and administered orally	Prostate, Cervical cancer
***Solanum nigrum* (L.)**		PRU0124972	Solanaceae	4	3.4%	Leaves, fruits, stem	Leaves and stems boiled and administered orally	Skin cancer, Prostate, Cervical cancer

### Preparation and Administration of Recipe

**Figure 2:**
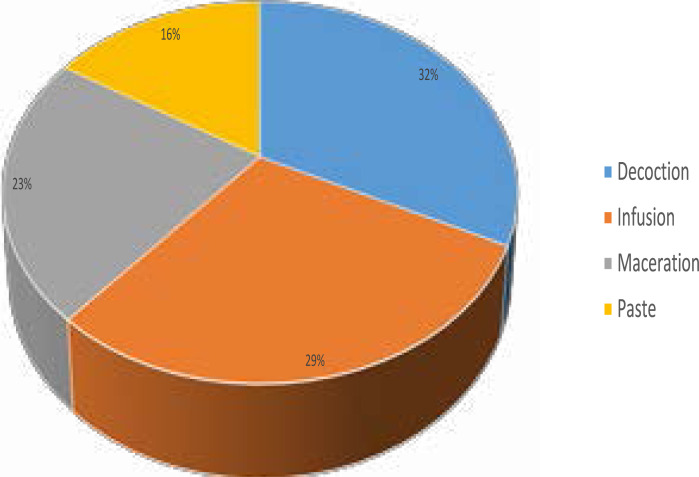
Methods of preparation of recipe

**Figure 3:**
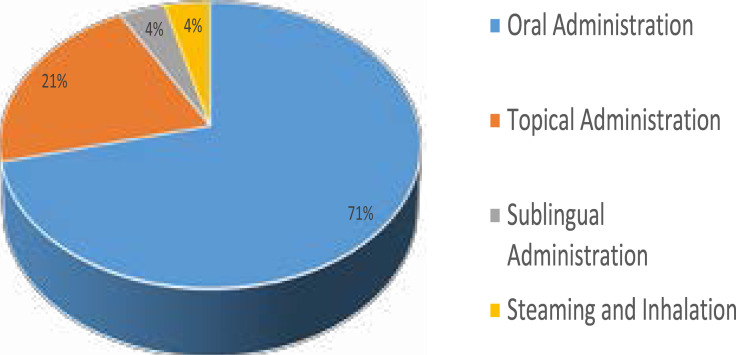
Administration of recipe

Decoction (32.3%) was the most preferred method of recipe preparation used by the traditional healers when compared to other three methods namely infusion, maceration and paste (Figure 2). This finding is in concordance with the report of Raez et al.^17^ where decoction was reported as the preferred method of recipe preparation in their study. Oral administration (75%) was the mostly used method by the traditional healers for treatment application as documented in this study (Figure 3). Maema et al.^15^ in their study of invasive plant species used for the treatment of diseases also recorded oral administration as the preferred method of medication on patients during treatment course. According to Yang and Ross^18^ in their study on the theories and concepts in the composition of Chinese herbal formulas, decoction with oral administration was reported to be absorbed quickly and have the strongest action among all the traditional types of recipe preparation and administration.

### Cancer prevalence and plant species with their families

High mortality rate from cancer among inhabitants of low-income countries is a great threat to the population growth as projected by the World Health Organization. Conservatively, over 26 million South Africans seek traditional medicine as their first choice of treatment^19^. The increased patronage of traditional herbal healing systems by patients is due to perceived limitations and reported side effects in conventional care^20^. This present study recorded twenty-eight (28) plant genera distributed in eighteen (18) different families (Table 1) in Hammanskraal and Winterveld areas of Gauteng Province, South Africa.

In this study, the most prevalent type of cancer treated by the traditional healers was skin cancer (Table 1). Skin cancer was one of the prevalent cancer type in South Africa and other countries like Australia, and New Zealand^19^. High radiation was the reason attributed to prevalence of skin cancer in the aforementioned countries^19^. The plants used for the treatment of skin cancer come from more than half of the reported families in this survey where leaves were the plant part mostly used.

Lung cancer is the next prominent type of cancer treated by the traditional healers as recorded in this survey (Table 1). Factors such as pollution from industries and lifestyle habits such as smoking have been suggested as the probable reason for lung cancer prevalence as recorded during this study. Lung cancer is the leading cause of death in Latin America and other developing countries that was attributable to people's smoking patterns in those countries^21^.

Plants recorded from this survey for breast cancer (third most common) treatment include; *Pittosporum viridiflorum* (Sims.), *Euphorbia ingens* E. Mey. ex Boiss., *Combretum mkuzense* J.D.Carr & Retief, *Vachellia karroo* (Hayne) Banfi & Galasso, *Trifolium pretense* L., *Justicia capensis* (Thunb.), and *Laggera decurrens* (Vahl) Hepper & J.R.I Wood (Table 1). Andrade Carli et al^22^, in their study on natural products with activities against multi-drug resistant tumor cells reported that *P. viridiflorum* caused lethality on MCF 7 cancer cells. This apoptotic ability of *P. viridiflorum* against MCF 7 cancer cell was attributed to the possession of some volatile and non-volatile compounds in the tissue of the plant^22^.

Prostate cancer was one of the least common cancers recorded in this study when compared to other cancer types (Table 1). Meanwhile, the plants documented in this study for the treatment of prostate cancer were *Hypoxis hemerocallidea* Fisch. & C.A. Mey and *Trifolium pratense* L. Hypoxis species efficacy on prostate cancer was attributed to the major constituents it contains (hypoxoside, and its active derivative rooperol)^23^. Different plant species belonging to the Pittosporaceae, Fabaceae, Asteraceae, Solanaceae, Acanthaceae, Apiaceae, Euphorbiaceae, Hyacinthaceae, and Alliaceae families have all been reported in literature for traditional use or mostly in vitro studies^23^–^28^.

## Conclusions

The practice of traditional medicine is an age long practice in South Africa and plays a critical role in healthcare management practices. This study documented plants that have been in use by the traditional healers for different cancer treatments from two areas in Tshwane Metropolitan Municipality. Literature have shown that a number of these documented plants are yet to undergo in vivo evaluation and clinical trials for cancer management. This study therefore increases the archive of documented plants for further in vivo investigation. Further studies on these documented plants are required and this should include research that will investigate the anticancer ability of these plants without compromising the immune system of the individuals when administered. Efficacy of the identified plant species in in vivo assays, followed by clinical trials where there are promising results is further required.
